# Association between LDL-R (exon 8 C.1171 G > A) polymorphisms and response to antiviral therapy in hepatitis C virus infection

**DOI:** 10.1038/s41598-026-38468-w

**Published:** 2026-02-21

**Authors:** Mohey Eldin Hassan Shikhoun, Hesham A. M. Ibrahim, Ahmed Abdou O. Abeed

**Affiliations:** 1 Analysis and Laboratories Department, Higher Technological Institute of Applied Health Sciences in Sohag, Ministry of Higher Education, Cairo, Egypt; 2 Department of Agricultural Zoology and Nematology, Faculty of Agriculture, Al Azhar University, Assiut Branch, Assiut, 71524 Egypt; 3 Department of Chemistry, Faculty of Science, Assiut University, Assiut, 71516, Egypt

**Keywords:** Hepatitis c virus, LDL-R (exon 8 C.1171 G/A), Genetic polymorphism, Direct-acting antiviral agents, Biomarkers, Diseases, Gastroenterology, Genetics, Immunology, Medical research, Microbiology, Molecular biology

## Abstract

The entry of the hepatitis C virus (HCV) into liver cells is closely associated with its interaction with the low-density lipoprotein receptor (LDL-R), which plays a crucial role in facilitating viral uptake. This study aimed to investigate the association between the LDL-R (exon 8 C.1171 G/A) gene polymorphism and response to antiviral therapy in patients infected with HCV. Participants were divided into three groups. Group I included 30 patients positive for both anti-HCV antibodies and HCV-RNA who did not respond to antiviral therapy. Group II consisted of 60 patients positive for anti-HCV but negative for HCV-RNA, indicating successful treatment response. and Group III comprised 50 healthy individuals negative for both anti-HCV antibodies and HCV-RNA, serving as controls. Diagnostic assessments included reverse transcriptase-polymerase chain reaction (RT-PCR), enzyme-linked immunosorbent assay (ELISA), and standard biochemical tests. Genotyping for LDL-R (exon 8 C.1171 G/A) polymorphisms was conducted using allele-specific PCR on patients from both the responder and non-responder groups. Of the 376 infected patients receiving antiviral therapy, 345 (91.8%) exhibited a positive response to treatment, whereas 31 (8.2%) did not. A total of 90 patients (60 responders and 30 non-responders) were included for genotypic analysis. Among responders, the A/A genotype of LDL-R (exon 8 C.1171 G > A) was the most prevalent (61.7%), whereas the G/G genotype was predominant among non-responders (76.7%). Genotyping analysis demonstrated that hepatitis C virus genotype 4 was the most common, being detected in all 20 responders and in 10 of 20 non-responders. These findings indicate a significant association between LDL-R (exon 8 C.1171 G/A) genetic variants and the response to antiviral therapy in Egyptian patients with chronic hepatitis C.

## Background

The hepatitis C virus (HCV) affects around 185 million people worldwide and significantly contributes to chronic liver disease, often progressing to liver cirrhosis and hepatocellular carcinoma (HCC)^1,2^. HCV is a single-stranded, positive-sense RNA virus classified within the Flaviviridae family and the Hepacivirus genus^3,4^. Increasing evidence demonstrates the intricate relationship between the HCV life cycle and host lipid metabolism, suggesting that HCV relies on lipid biosynthesis, lipoviral particles assembly, and lipid droplets for entry, secretion, and replication. Chronic HCV infection disrupts host lipid homeostasis, enhancing dyslipidemia, hepatic steatosis, and altered lipoprotein profiles, while HCV virions themselves circulate as lipid-enriched particles that use lipoprotein receptors for hepatocyte entry and intracellular propagation^5^. Egypt has historically had the highest HCV prevalence worldwide. The national anti-HCV seroprevalence rate among people aged 15 to 59 is 14.7%, which is much higher than in any other country^6^. HCV can infect various cell types; however, hepatocytes are the principal site of viral replication^7,8^. The exact mechanisms by which HCV attaches to and enters hepatocytes remain unclear. Several in vitro models have been used to investigate HCV entry, including HCV-LP expressed in insects, VSV/HCV pseudotype systems, and retroviral pseudotyped particles (HCVpp)^9,10^. More recently, cell culture-derived infectious HCV systems (HCVcc), especially those based on Huh-7 cells, have made it possible to better understand viral receptors^9^. Hepatitis C Virus entry is a complex and highly regulated multistep process involving several host receptors and co-factors^11^. Multiple cellular receptors have been identified as potential mediators of HCV entry, involving the low-density lipoprotein receptor (LDLR)^12^, Asialoglycoprotein receptor, DC-SIGN and L-SIGN, scavenger receptor-B type I, and CD81, and facilitating viral attachment and concentration in hepatic tissue^13,14^.

LDL-R is of particular interest because it facilitates entry of some rhinoviruses and maintains cholesterol homeostasis^15,16^. Genetic mutations and polymorphisms in the LDL-R gene have also been associated with disorders like familial hypercholesterolemia, atherosclerosis, and obesity. A significant correlation has been observed between LDL-R expression and intracellular HCV RNA levels, both in vivo and in cultured hepatocytes, suggesting that LDL-R functions as an essential co-receptor for HCV entry and replication^17^. Given its immunological relevance and the effects of genetic variations on disease susceptibility, LDL-R (exon 8 C.1171 G/A) is a compelling candidate for studying host genetic factors affecting antiviral treatment response^18^. Therefore, the present study investigates the potential association between LDL-R (exon 8 C.1171 G/A) gene polymorphisms and treatment response in Egyptian patients with chronic HCV infection, aiming to elucidate whether genetic variation in this receptor influences therapeutic outcomes.

## Materials and methods

### Ethics statement

Written informed consent was obtained from each participant. Blood samples were collected during routine clinic appointments for various assays. The patients agreed to the treatment plans and registration requirements for the National Treatment Program that were provided by the Ministry of Health. 90 HCV patients, unaware of their LDL-R gene polymorphisms, were randomly enrolled in the study. The protocol was reviewed and formally approved by the Faculty of Science Research Ethics Committee (FSREC) under protocol number [01-25025-0011]. All methods were carried out in strict accordance with the relevant human-subject research guidelines and regulations, including adherence to institutional policies of Tahta Hospital, the national ethical standards for biomedical research in Egypt, and international principles outlined in the Declaration of Helsinki and Good Clinical Practice guidelines.

### Study design and patients

This observational case–control study was conducted on 90 Egyptian patients with chronic hepatitis C virus (HCV) infection. Patients were recruited from Tahta Hospital, Egypt, and classified as 60 responders and 30 non-responders to direct-acting antiviral (DAA) therapy. Responders were patients who achieved sustained virological response (SVR), defined as undetectable HCV RNA 12 weeks post-therapy. Non-responders were defined as patients who did not achieve SVR or showed detectable HCV RNA at the same time point. Baseline characteristics recorded for all participants included age, sex, HCV genotype, baseline viral load, and treatment regimen, to describe the study population.

### Inclusion and exclusion criteria

The study included adult patients aged 18 years or older with confirmed chronic HCV infection who received standard DAA therapy according to current clinical guidelines, completed the full prescribed treatment course, and had complete clinical, demographic, and virological data available for analysis. Patients were excluded if they had co-infection with hepatitis B virus (HBV) or human immunodeficiency virus (HIV), decompensated liver disease, hepatocellular carcinoma, or other significant comorbidities that could affect treatment response, had received previous antiviral treatment for HCV before the current therapy, or had incomplete or missing clinical, laboratory, or virological data.

### Collection and processing of blood samples

A total of 3,450 individuals participated in a rapid screening study for HCV, employing an ELISA technique to detect anti-HCV antibodies. A PCR-based method was modified to detect genetic variation in the LDL-R gene. Blood samples were obtained through a venipuncture of the cubital vein. The collection site was thoroughly cleaned with 70% isopropyl alcohol, then a 1% iodine solution was applied and allowed to dry. Five milliliters of blood were drawn with a sterile syringe and transferred into a clean plastic container to avoid contamination. The samples underwent centrifugation at 4,000 rpm for 10 min to isolate the serum, which was then preserved at -70 °C for subsequent analysis. The screening results indicated that 376 out of 3,450 individuals (10.9%) tested positive for HCV antibodies, while 3,074 (89.1%) were seronegative. Among the 376 antibody-positive individuals, following sofosbuvir-based antiviral therapy, 345 (91.8%) exhibited a favorable response, whereas 31 patients (8.2%) did not respond to the treatment. Researchers selected 30 non-responders and 60 responders for further analysis after the six-month treatment period. Blood samples were collected during routine clinical visits and analyzed using various laboratory techniques. The study population was categorized into three groups: Group I included 30 patients who were positive for both anti-HCV antibodies and HCV-RNA and failed to achieve sustained virological response (SVR) following antiviral therapy; Group II consisted of 60 patients who were anti-HCV positive and achieved SVR, defined as undetectable HCV RNA by PCR 12 weeks after completion of antiviral therapy; and Group III comprised 50 healthy individuals who tested negative for both anti-HCV antibodies and HCV-RNA, serving as normal controls.

### Serum markers for HCV infection

Third-generation ELISA assays were employed to identify anti-HCV antibodies. The microplate wells were coated with recombinant antigens that represent essential epitopes from the HCV NS3, NS4, and NS5 regions. The color reaction intensity was directly proportional to the concentration of anti-HCV antibodies present in the sample. The assay utilized the BioKit-bioelisa HCV 4.0 ELISA kit.

### Liver function test

The liver function tests were conducted using the Spectrophotometer system (Photometer 5010, manufactured by Robert Riele GMbH & Co. KG, German). Liver enzymes (ALT, AST, ALP, GGT and LDH) were measured using standard enzymatic colorimetric methods according to the manufacturer’s instructions. The assays are based on enzyme-catalyzed reactions producing a colorimetric change proportional to enzyme activity, which was measured spectrophotometrically at specific wavelengths. The reagents used for each test were provided by Human Diagnostic Worldwide, a Germany-based company.

### Alpha Fetoprotein (AFP) tumor marker test

Through employing a sandwich immunodetection method, the antibody immobilized on the test strip forms antigen-antibody complexes by binding to the antigen in the sample. The detector antibody present in the buffer captures these complexes as they migrate along the nitrocellulose membrane. As the antigen concentration increases, more antigen-antibody complexes form, resulting in a stronger fluorescence signal from the detector antibody. This signal is then analyzed by the iChromTM device to quantify the level of alpha-fetoprotein (AFP) in the sample. The detection buffer is first added to a tube, followed by 30 µL of whole blood; the mixture is gently swirled approximately ten times. Then, 75 µL of the prepared sample is dispensed into the sample well of the test cartridge and incubated at room temperature for 15 min. The ichroma™ device is used to read and interpret the results.

## Molecular diagnostic

### HCV RNA extraction

Viral RNA was extracted using the QIAamp Viral RNA Mini Kit (QIAGEN, Hilden, Germany; Cat. No. 59906) according to the manufacturer’s instructions. Briefly, HCV RNA was eluted in a final volume of 60 µL of AVE buffer and stored at − 80 °C until further molecular analysis.

### Qualitative polymerase chain reaction (PCR)

Complementary DNA (cDNA) synthesis was performed from extracted HCV RNA using gene-specific primers (forward: 5′-ACCCTCGTTTCCGTACAGAG-3′; reverse: 5′-CGCGCGACTAGGAAGACTTC-3′) in a two-step RT-PCR protocol. In the first step, reverse transcription was conducted at 42 °C for 50 min to synthesize cDNA, followed by inactivation at 95 °C for 5 min. The second step involved PCR amplification for 40 cycles, comprising denaturation at 94 °C for 30 s, annealing at 57 °C for 30 s, and extension at 72 °C for 30 s, followed by a final extension at 72 °C for 5 min. This two-step approach enhances both the sensitivity and specificity of HCV detection, particularly for samples with low viral loads.

### LDL-R (exon 8 C.1171 G/A) genotype polymorphism

#### Preparation of peripheral blood mononuclear cells (PBMC)

Five milliliters of whole blood were collected from each participant and placed in EDTA-containing anticoagulant tubes. Under aseptic conditions, 2.5 mL of Ficoll-hypaque density gradient separating solution was carefully layered along the side wall of a centrifuge tube containing a blood sample (5 mL). The mixture was centrifuged at 2,000 rpm for 20 min at room temperature, resulting in the separation of blood components into four distinct layers. The top layer, consisting of plasma and platelets, was carefully aspirated and stored in sterile tubes at -80 °C for subsequent antibodies and biochemical analyses. Peripheral blood mononuclear cells (PBMC) formed a distinct layer at the interface between the plasma and Ficoll-hypaque, with a density of approximately 1.77 g/mL. The bottom layer consisted of erythrocytes. PBMCs were isolated from the anticoagulated blood of each participant. The samples were diluted 1:1 with Dulbecco’s PBS and PBMC were separated using a lymphocyte separation medium (LSM). The leucocyte-rich interphase was washed with PBS, centrifuged, and the resulting PBMC pellet was stored in sterile tubes at -80 °C until further use for LDL-R gene amplification. After initial leukocyte separation using Ficoll-Hypaque, a second purification step was performed using a lymphocyte separation medium (LSM) to enhance the purity of PBMCs, ensuring removal of residual erythrocytes and platelets for downstream DNA extraction and genotyping.

#### DNA extraction from PBMCs

DNA was extracted from the PBMC fraction using the QIAamp DNA Mini Kit (QIAGEN, Hilden, Germany; Cat. No. 51304), following the manufacturer’s spin-column protocol. Briefly, PBMCs were lysed and treated with proteinase K, followed by binding to the silica membrane, washing, and elution in 200 µL AE buffer. This protocol ensured high-quality genomic DNA suitable for downstream LDL-R genotyping.

#### LDL-R (exon 8 C.1171 G/A) genotypes determination

Genotyping of LDL-R allelic variations was conducted using real-time PCR with the QuantiTect^®^ Probe PCR Kit (Qiagen, Hilden, Germany) on a StepOne Real-Time PCR system. Each 15 µL reaction mixture contained 1× reaction buffer, 1–2 mmol/L MgCl₂, 0.32 mmol/L dNTPs, 0.12 µmol/L of both forward and reverse primer, and allele-specific probes. The primer sequences were forward primer 5’-GTTGCAGAAAGTGTAAAAATTAGTA-3’ and reverse primer 5’-TAACCTCATTCAGGACTTCC-3’. The allele-specific probes included the allele 1 probe (VIC-ACACGAAGGCCTGC-NGR) and the allele 2 probe (6-FAM-ACACGAAGACCTGC-NFQ). The reaction also contained 1 unit of Taq polymerase, 50 ng of genomic DNA, and nuclease-free water (dH₂O). Thermal cycling conditions began with an initial incubation at 50 °C for 2 min, followed by denaturation at 95 °C for 15 min, and then 40 amplification cycles consisting of 15 s at 95 °C and 1 min at 60–64 °C. After amplification, 1 µL of each LDR product was analyzed using an ABI Prism 310 Genetic Analyzer (Applied Biosystems, Warrington, UK). Genotyping was performed with Genotyper software programs (Applied Biosystems).

### DNA sequencing

PCR products were sequenced in both forward and reverse directions using the BigDye™ Terminator v3.1 Cycle Sequencing Kit (Applied Biosystems, Foster City, CA, USA) following the manufacturer’s protocol. The sequencing reaction was prepared in a final volume of 20 µL, comprising 8 µL of Big Dye terminator mix, 3.2 µL of 1 pmol diluted forward or reverse primer, 1 µL of purified PCR product, and 7.8 µL of nuclease-free water. Thermal cycling was performed for 25 cycles with the following parameters: initial denaturation at 94 °C for 4 min, followed by 95 °C for 15 s, 55 °C for 30 s, and 60 °C for 4 min. The amplified sequencing products underwent purification via Centri-sep™ nucleic acid gel-filtration columns (Invitrogen, Life Technology). Ten microliters of purified PCR products were combined with ten microliters of Hi-Di™ formamide, denatured at 95 °C for 5 min, and sequenced using an ABI PRISM^®^ 310 Genetic Analyzer (Applied Biosystems, Foster City, CA, USA). Sequence analysis was performed using both forward and reverse chromatograms to ensure data accuracy. Sequences were compared with GenBank reference sequences, and alignment/editing was conducted using BioEdit software.

### HCV genotyping

HCV genotypes were identified by direct sequencing of the non-structural (NS5B) viral gene using universal primers. Amplification was performed by a one-step RT-PCR kit (QIAGEN, Inc.). The primers were sense 5’-TTCTCRTATGAYACCCGCTGYTTTGA-3’ and antisense 5’-TACCTVGTCATAGCCTCCGTGAA-3’. The primers used for amplification of the NS5B region were previously validated in genotyping studies^51^.

### DNA purification and sequencing

The PCR amplicons were purified utilizing the innuPREP PCRpure kit (Analytik Jena, Germany) and subsequently sequenced employing the dideoxynucleotide termination method with the ABI PRISM^®^ BigDye Terminator Cycle Sequencing kit (Applied Biosystems, Foster City, CA, USA). Sequence analysis was conducted using an ABITM 3130 Genetic Analyzer (Applied Biosystems, Foster City, CA, USA). The sense and antisense primers were utilized as sequencing primers as well. Chromatogram files were analyzed in Chromas 2.3 (Technelysium, Helensvale, Australia), and the genetic makeup of each sample was established by comparing its sequence to HCV prototype sequences obtained from GenBank.

### Phylogenetic analysis

Phylogenetic relationships among HCV isolates were assessed using the neighbor-joining method with 1,000 bootstrap replicates in MEGA version 4.0.1. Sequences were aligned using ClustalW, and evolutionary distances were calculated using the Kimura 2-parameter model. Bootstrap values were reported to indicate the reliability of each branch in the phylogenetic tree.

### Statistical analysis

Statistical analyses were performed using SPSS version 25.0. Continuous variables were summarized as mean ± standard deviation (SD), while categorical variables were presented as counts and percentages. One-way analysis of variance (ANOVA) was used to compare continuous variables among groups, followed by post-hoc comparisons using the least significant difference (LSD) method. Chi-square or Fisher’s exact test was used for categorical variables as appropriate. Allele frequencies for LDL-R (exon 8 C.1171 G/A) polymorphism were calculated by direct counting. The genotype distribution (AA, GA, GG) was tested for compliance with Hardy-Weinberg Equilibrium using the chi-square (χ²) test. A P-value greater than 0.05 was considered consistent with equilibrium. P-values < 0.05 were considered statistically significant, and all tests were two-tailed.

## Results

### Detection of anti-HCV antibody and HCV RNA

**Fig. 1:**
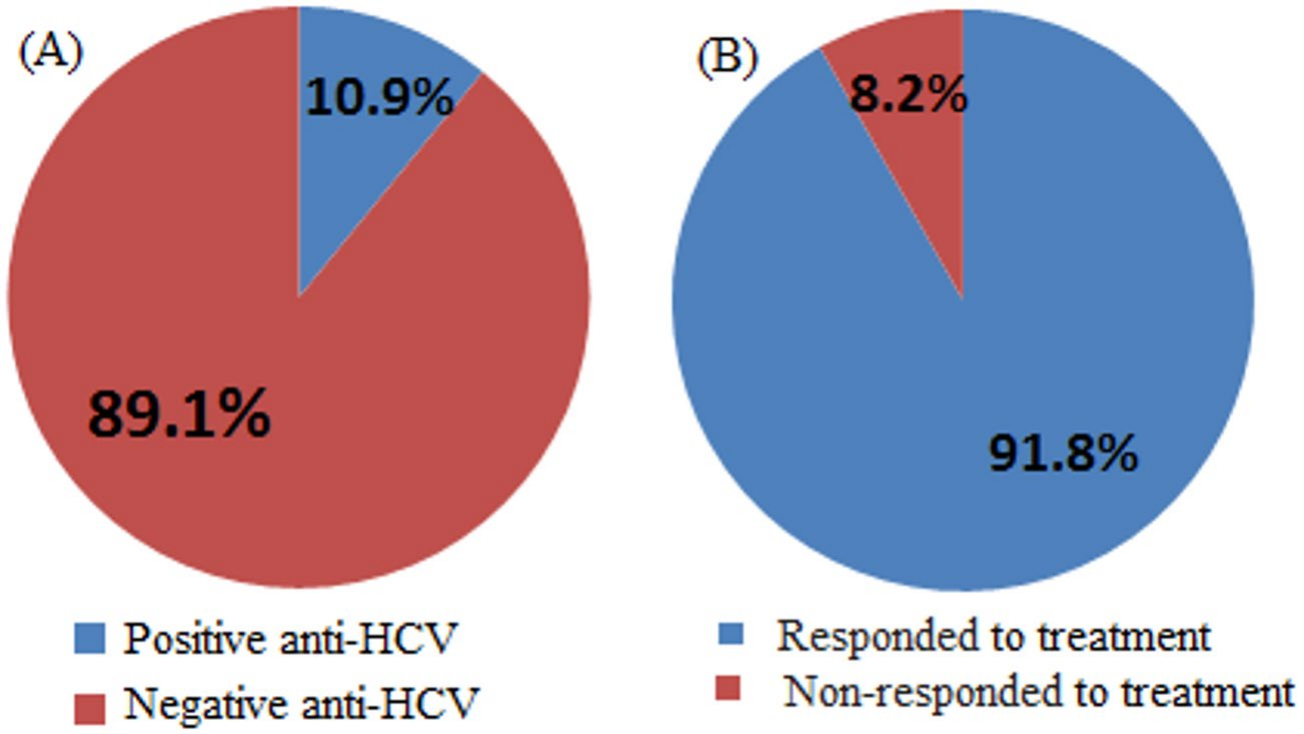
**(A)** Display the HCV infection rate for each sample that was collected. **(B)** show the percentage of responded and non-responded after treatment to sofosbuvir.

Three thousand four hundred and fifty individuals were screened for hepatitis C virus using the ELISA technique for anti-HCV antibody detection. Our findings indicated that out of 3450 individuals, 376 (10.9%) were positive for hepatitis C virus antibodies and 3074/3450 (89.1%) individuals negative for hepatitis C antibodies by ELISA technique for anti-HCV antibody detection (Table 1; Fig. 1a).

### Virological findings

#### Detection of anti-HCV antibody and HCV RNA

Our findings showed that among the 376 HCV-infected patients treated with sofosbuvir-based therapy, 345 patients (91.8%) achieved a sustained virological response (SVR), defined as undetectable HCV RNA 12 weeks after completion of treatment, whereas 31 patients (8.2%) failed to achieve SVR and were classified as non-responders (Table 2; Fig. 1b).

**Table 1:** ELIZA test for anti-HCV detection on all collected samples.

No.patients	Results	Percent %
376	Positive anti-HCV	10.9%
3074	Negative anti-HCV	89.1%

**Table 2:** HCV RNA molecular detection for every patient following therapy.

No. of patients	Percent (%)	Responder to antiviral therapy	HCV RNA by PCR
345	91.8%	Responded	Negative
31	8.2%	Non-responded	Positive

### Biochemical analysis

#### Biochemical analysis of responders and non-responders regarding biochemical analysis after treatment with antiviral drug

**Fig. 2:**
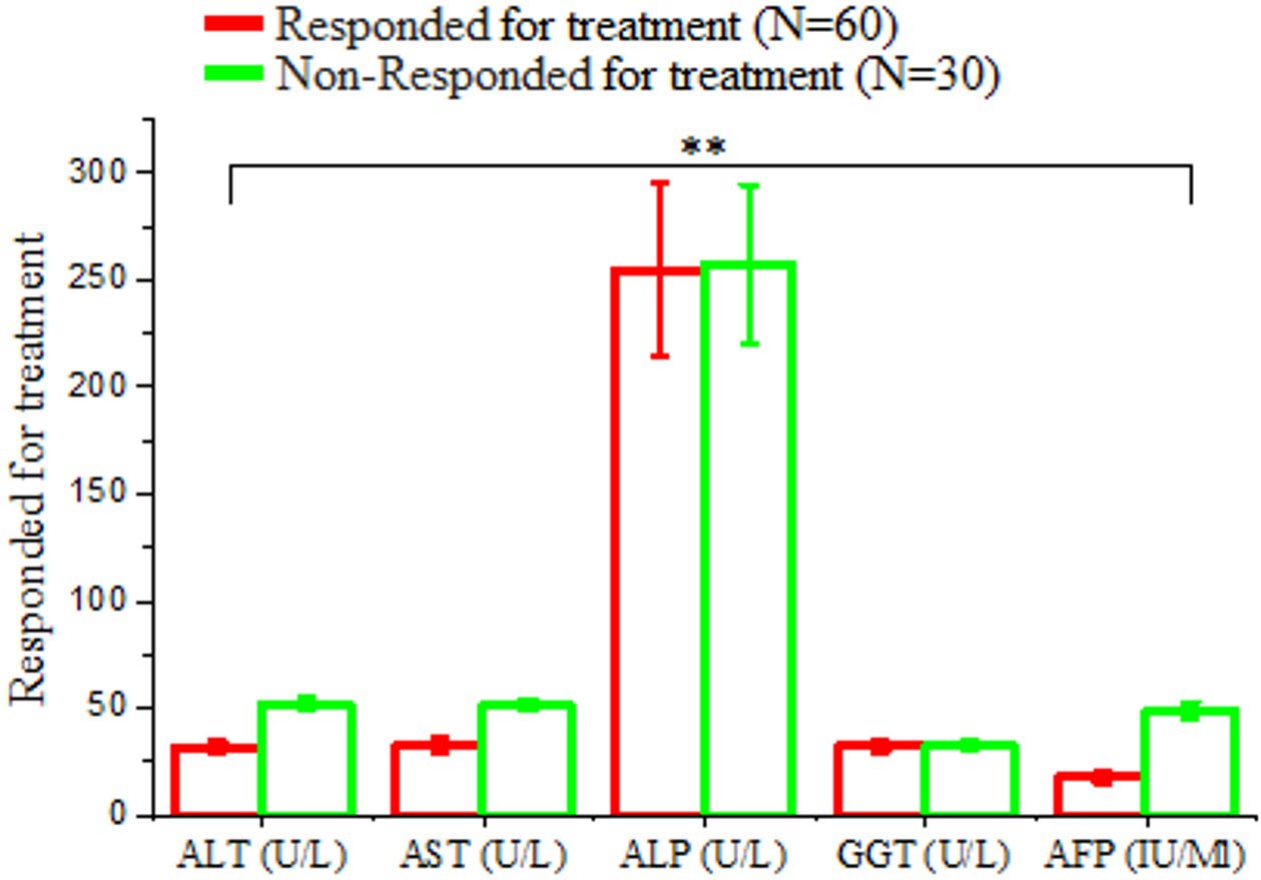
Comparison of liver function biomarkers between HCV treatment responders (*N* = 60) and non-responders (*N* = 30). The bar chart illustrates mean ± SD values for ALT, AST, ALP, GGT, and AFP levels. A statistically significant difference (*P* < 0.05) were observed in ALT, AST and AFP, while other parameters showed no significant differences.

Presents a comparison of liver function biomarkers between patients who responded to sofosbuvir-based antiviral therapy (n = 60) and those who did not respond (n = 30). The mean ALT level was lower in responders (31.92 ± 2.68 U/L) compared to non-responders (51.95 ± 2.91 U/L), with a highly significant difference (P = 0.000; HS) (Tables 3 and Fig. 2).

**Table 3:** Comparison of liver function test between responders and non-responders following sofosbuvir –based antiviral therapy.

Variables		Responded for treatment (*N* = 60)	Non-Responded for treatment (*N* = 30)	T. test ^a^	*P*-value	Sig.
ALT (U/L)	Mean ± SD	31.92 ± 2.68	51.95 ± 2.91	-22.24	0.000	H.S
AST (U/L)	Mean ± SD	32.71 ± 2.96	51.45 ± 2.67	-21.29	0.000	H.S
ALP (U/L)	Mean ± SD	254.57 ± 39.9	257.14 ± 37.04	-0.838	0.434	N.S
GGT (U/L)	Mean ± SD	32.17 ± 2.84	32.64 ± 2.58	-1.034	0.341	N.S
AFP (IU/Ml)	Mean ± SD	17.97 ± 2.76	48.87 ± 3.89	-16.91	0.000	H.S

N.S: Non-Significant H.S: Highly Significant

#### Biochemical analysis responder after treatment with antiviral drug and control

**Fig. 3:**
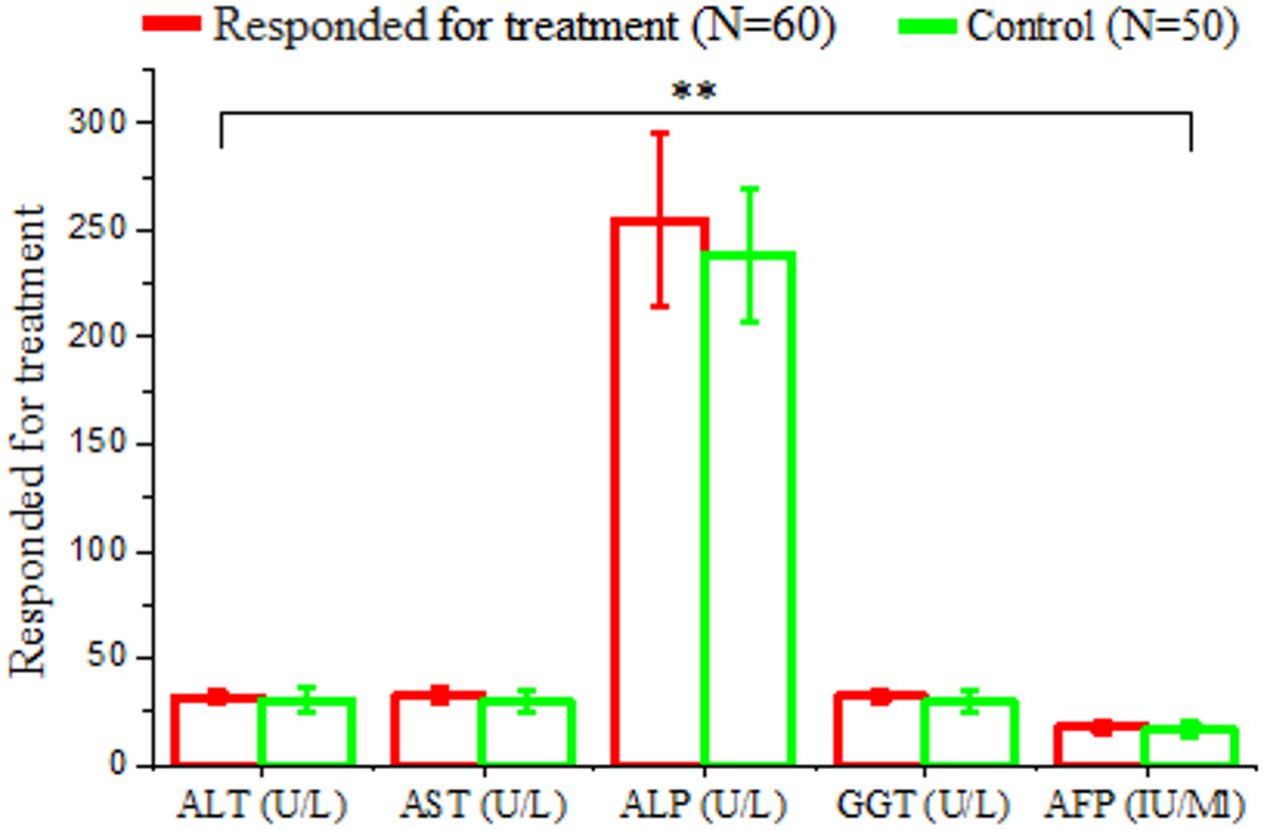
Comparison of liver function markers between HCV treatment responders (*N* = 60) and healthy controls (*N* = 50). Bar graph represents mean ± SD for ALT, AST, ALP, GGT, and AFP levels. A non -significant difference were observed in all parameters between the two groups (*P* < 0.05; NS).

The comparison of liver function biomarkers between patients who responded to sofosbuvir-based antiviral therapy (*n* = 60) and healthy control subjects (*n* = 50) is illustrated in Table 4. The ALT level in responders (31.92 ± 2.68 U/L) was higher than in controls (30.46 ± 5.60 U/L), with no statistically significant difference observed (*P* = 0.637; NS). AST levels were slightly higher in responders (32.71 ± 2.96 U/L) compared to controls (29.67 ± 5.25 U/L), though this difference was not of statistical significance (*P* = 0.158; NS) (Table 4 and Fig. 3).

**Table 4:** Comparison of liver function test result between responders following Sofosbuvir –based antiviral therapy and healthy controls.

Variables		Responded for treatment (*N* = 60)	Healthy controls (*N* = 50)	T. test ^a^	*P*-value	Sig.
ALT (U/L)	Mean ± SD	31.92 ± 2.68	30.46 + 5.60	0.637	0.637	N.S
AST (U/L)	Mean ± SD	32.71 ± 2.96	29.67 + 5.25	1.610	0.158	N.S
ALP (U/L)	Mean ± SD	254.57 ± 39.9	238.15 + 31.76	1.332	0.231	N.S
GGT (U/L)	Mean ± SD	32.17 ± 2.84	29.68 + 5.05	1.276	0.249	N.S
AFP (IU/Ml)	Mean ± SD	17.97 ± 2.76	16.46 + 3.25	1.356	0.224	N.S

N.S: Non-Significant

#### Biochemical analysis of non-responder after treatment with antiviral drug and control

**Fig. 4:**
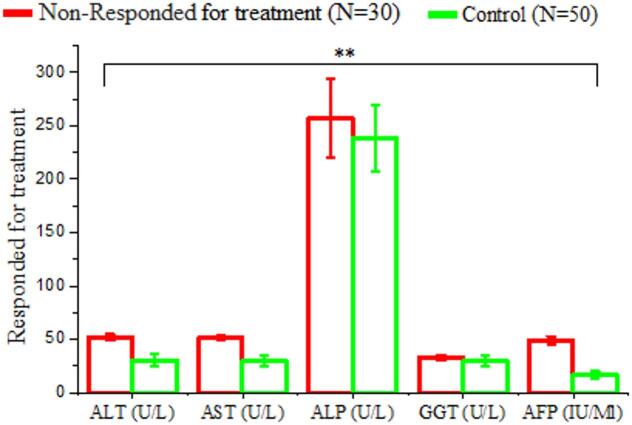
Comparison of liver function biomarkers between HCV treatment non-responders (*N* = 60) and healthy controls (*N* = 50). The bar graph shows mean levels (± SD) of ALT, AST, ALP, GGT, and AFP. A statically significant difference (*P* < 0.01; HS) were observed in ALT, AST, and AFP except ALP and GGT showed no remarkable different between the two groups.

Presents a comparative assessment of liver function parameters between patients who did not respond to sofosbuvir-based antiviral therapy (non-responders, n = 30) and healthy control subjects (n = 50). The data reveal significant differences in key hepatocellular markers, exhibiting ongoing liver injury in the non-responder group. ALT levels were substantially higher in non-responders (51.95 ± 2.91 U/L) than controls (30.46 ± 5.60 U/L), with a highly significant difference (P = 0.000; HS). AST levels were markedly higher in non-responders (51.45 ± 2.67 U/L) than in controls (29.67 ± 5.25 U/L), also reaching high statistical significance (P = 0.000; HS) (Tables 5 and Fig. 4).

**Table 5:** Comparison of liver function test result between non-responders following sofosbuvir –based antiviral therapy and healthy controls.

Variables		Non-responded for treatment (*N* = 30)	Healthy controls (*N* = 50)	T. test ^a^	*P*-value	Sig.
ALT (U/L)	Mean ± SD	51.95 ± 2.91	30.46 + 5.60	9.218	0.000	H.S
AST (U/L)	Mean ± SD	51.45 ± 2.67	29.67 + 5.25	9.273	0.000	H.S
ALP (U/L)	Mean ± SD	257.14 ± 37.04	238.15 + 31.76	1.602	1.602	N.S
GGT (U/L)	Mean ± SD	32.64 ± 2.58	29.68 + 5.05	1.444	1.990	N.S
AFP (IU/Ml)	Mean ± SD	48.87 ± 3.89	16.46 + 3.25	21.541	0.000	H.S

N.S: Non-Significant H.S: Highly Significant

### LDL-R (exon 8 C.1171 G/A) genetic polymorphism by PCR

#### LDL-R (exon 8 C.1171 G/A) gene polymorphism for both responder and non-responder after antiviral therapy

**Fig. 5:**
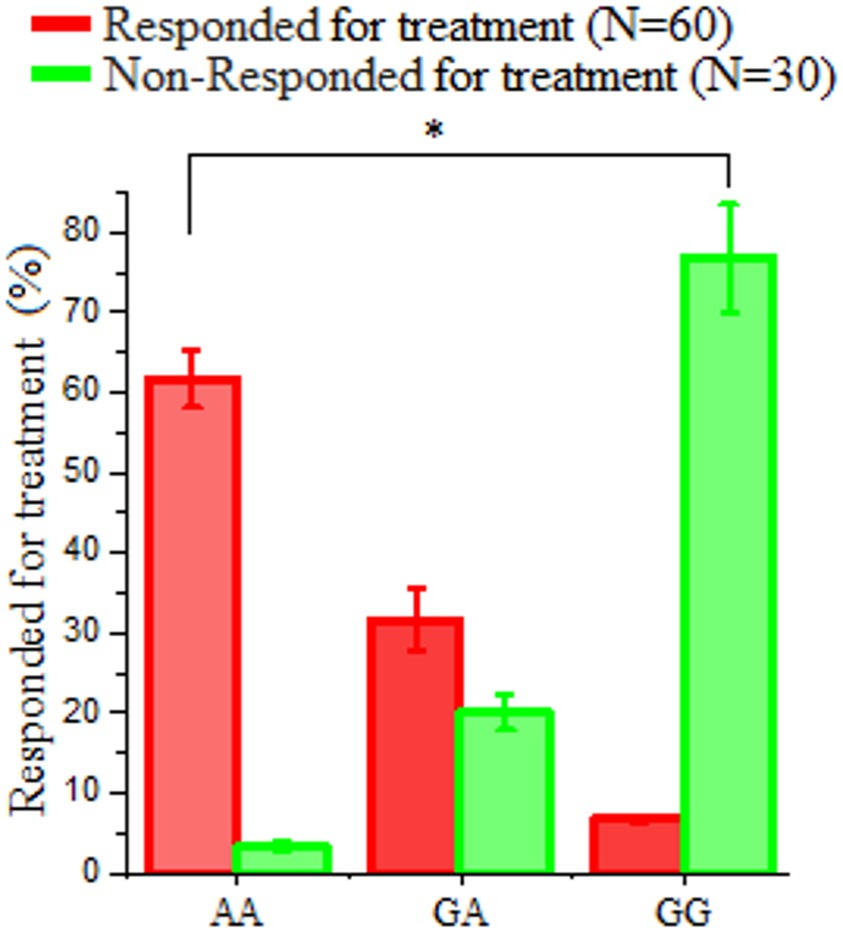
Treatment response rates by LDL-R (exon 8 C.1171 G/A) genotype in hepatitis C patients. Responders (*N* = 60) showed highest response rates with AA genotype (61.7%), followed by GA (31.6%) and GG (6.7%) genotypes. Non-responders (*N* = 30) demonstrated an inverse pattern, with highest non-response in GG genotype (76.7%). This variation in genotype frequencies was statistically significant (χ² = 49.765, *P* < 0.000; highly significant).

As presented in Table 6, the distribution of LDL-R (exon 8 C.1171 G/A) gene polymorphisms (AA, GG, and GA genotypes) significantly differed between the responder and non-responder groups following antiviral therapy. Among the responders (*n* = 60), the AA genotype was predominant 61.7% of individuals, followed by GA in 31.6%, and GG in only 6.7% (Table 6 and Fig. 5).

**Table 6:** Distribution of LDL-R (exon 8 C.1171 G/A) gene polymorphism among responders and non-responders following antiviral therapy.

Variables	Responded to treatment (*N* = 60)	Non-responded to treatment (*N* = 30)	X^2^	*p* value
AA	37 (61.7%)	1 (3.3%)	49.765	0.000 H.S
GA	19 (31.6%)	6 (20%)		
GG	4 (6.7%)	23 (76.7%)		

H.S: Highly Significant.

##### LDL-R (exon 8 C.1171 G/A) gene polymorphism for both responder after antiviral therapy and control

**Fig. 6:**
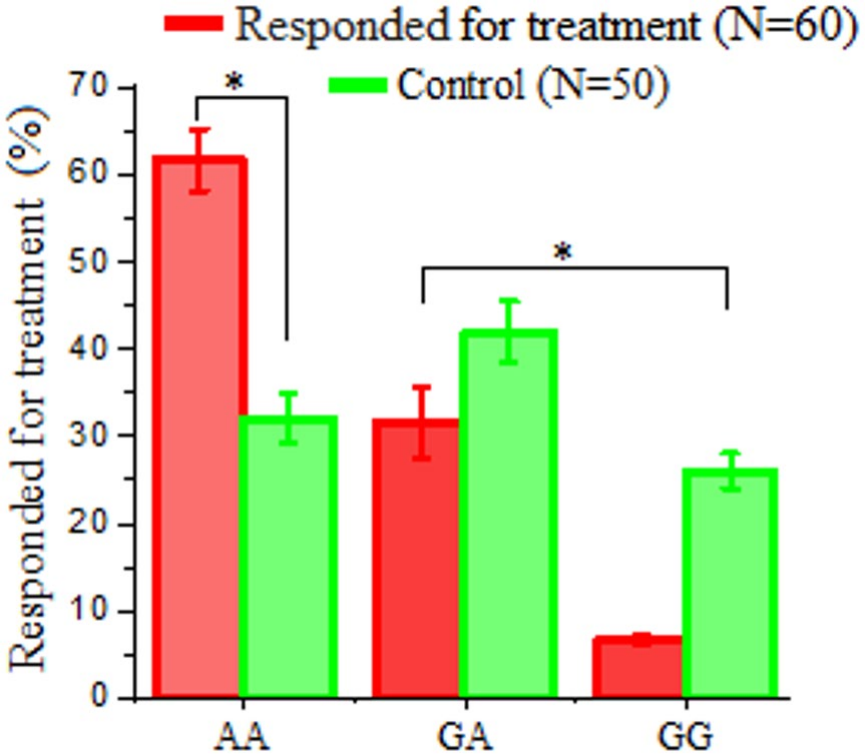
Distribution of LDL-R (exon 8 C.1171 G/A) genotypes (AA, GA, GG) among hepatitis C patients who responded to treatment (*n* = 60) compared to controls (*n* = 50). The AA genotype was most prevalent in responders (61.7%), while controls showed a more balanced genotype distribution (AA: 32%; GA: 42%; GG: 26%). The difference in genotype frequencies between groups was statistically significant (χ² = 12.378, *P* < 0.000; highly significant), suggesting a potential association between the AA genotype and favorable treatment response. Error bars represent standard deviation.

As presented in Table 7, a significant difference was obtained in the distribution of LDL-R (exon 8 C.1171 G/A) gene polymorphisms (AA, GA, and GG genotypes) between the responder group (*n* = 60) and the control group (*n* = 50). The AA genotype was predominant (61.7%), followed by GA (31.6%) and GG (6.7%). The control group indicated a more balanced distribution: 32% AA, 42% GA, and 26% GG (Table 7 and Fig. 6).

**Table 7:** Distribution of LDL-R (exon 8 C.1171 G/A) gene polymorphism among responder patients following antiviral therapy and healthy controls.

Variables	Responded to treatment (*N* = 60)	Healthy controls (*N* = 50)	X^2^	. *p*.value
AA	37 (61.7%)	16 (32%)	12.378	0.000 H.S
GA	19 (31.6%)	21 (42%)		
GG	4 (6.7%)	13 (26%)		

H.S: Highly Significant.

#### LDL-R (exon 8 C.1171 G/A) gene polymorphism for both non-responder after antiviral therapy and control

**Fig. 7:**
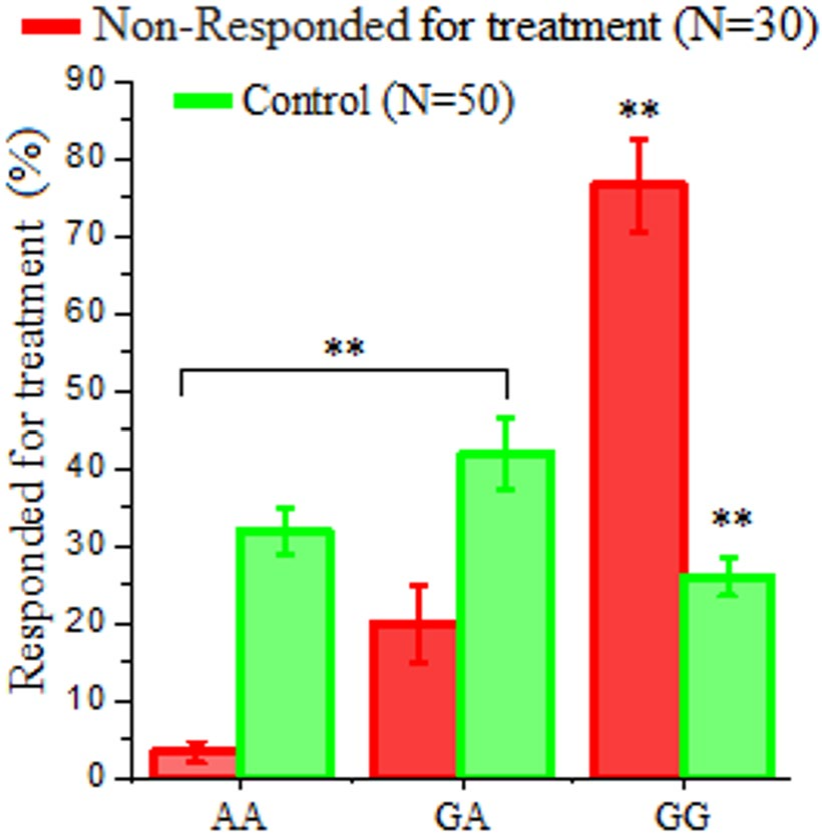
Distribution of LDL-R (exon 8 C.1171 G/A) genotypes (AA, GA, GG) among hepatitis C treatment non-responders (*N* = 30) compared to healthy controls (*N* = 50). The GG genotype was markedly overrepresented in non-responders (76.7%) compared to controls (26%), while the AA genotype was rare in non-responders (3.3%) versus controls (32%) and the GA genotype in non-responders (20%) versus controls (42%). The analysis showed a highly significant difference in genotype frequencies between the two groups (χ² = 20.636, *P* < 0.000; highly significant).

As presented in Table 8, a significant difference was observed in the distribution of LDL-R (exon 8 C.1171 G/A) gene polymorphisms (AA, GA, and GG genotypes) between the non-responder group (*n* = 30) and the control group (*n* = 50). The AA genotype was predominant (3.3%), followed by GA (20%) and GG (76.7%). The control group indicated a more balanced distribution. Figure 7 illustrated the distribution of LDL-R (exon 8 C.1171 G/A) gene polymorphisms (AA, GA, and GG genotypes) in patients who failed to respond to sofosbuvir-based antiviral therapy (*n* = 30) compared to a healthy control group (*n* = 50). Among non-responders, the GG genotype was predominant, found in 76.7% of patients, followed by GA (20%) and AA (3.3%). Sequentially, the control group indicated a more balanced distribution: 32% AA, 42% GA, and 26% GG (Table 8 and Fig. 7**).**

**Table 8:** Distribution of LDL-R (exon 8 C.1171 G/A) gene polymorphism among non-responder patients following antiviral therapy and healthy controls.

Variables	Non-responded to treatment (*N* = 30)	Healthy controls (*N* = 50)	X^2^	*p *value
AA	1 (3.3%)	16 (32%)	20.636	0.000 H.S
GA	6 (20%)	21 (42%)		
GG	23 (76.7%)	13 (26%)		

H.S: Highly Significant.

### HCV genotyping for responder and non-responder after antiviral therapy

**Fig. 8:**
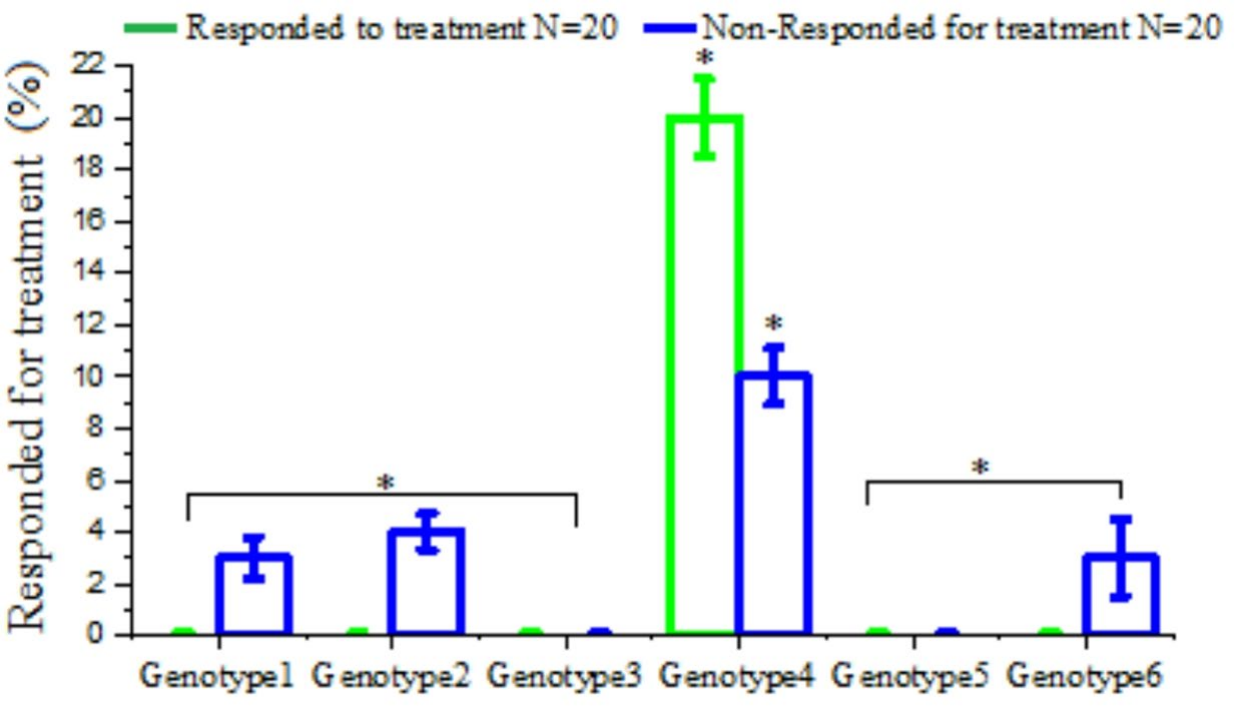
Distribution of HCV genotyping for both responder and non-responder after antiviral therapy.

Forty samples were randomly selected from different individuals to determine the HCV genotype prior to initiating antiviral therapy. Of these, twenty samples were taken from individuals who had not responded to antiviral therapy, and twenty were from individuals who had responded successfully. Our analysis revealed that among the responders, 20 out of the samples were identified as HCV genotype 4. Similarly, among the non-responders, 10 out of 20 samples were genotype 4, while 3 were genotype 1, 4 were genotypes 2, and 3 were genotype 6 (Fig. 8).

## Discussion

A total of 3,450 individuals were screened for HCV using ELISA. Of these, 376 (10.9%) were positive and 3,074 (89.1%) negative, highlighting the need for ongoing surveillance, early diagnosis, and effective therapy^19,20^. Among the HCV-positive patients treated with sofosbuvir, 345 (91.8%) achieved SVR, while 31 (8.2%) did not. Host factors, including LDL-R polymorphisms, baseline viral load, viral genotype, liver fibrosis, and adherence, may influence treatment response^21,22^. Responders had lower ALT and AST compared to non-responders, although ALT alone may not reliably predict response. AST elevations in non-responders likely reflect more extensive hepatocellular injury or fibrosis rather than serving as definitive predictors^23,24^. ALP and GGT levels were comparable between responders and non-responders, consistent with a hepatocellular pattern of injury. AFP was significantly higher in non-responders, suggesting greater hepatic stress or progression toward advanced liver disease^26^; however, it should be interpreted as an associated marker rather than a definitive predictor^25,27,28^. Compared to healthy controls, responders showed near-normalization of ALT, AST, ALP, GGT, and AFP, consistent with resolution of hepatocellular injury following SVR^29,30^. Slightly higher levels of ALT, AST, and AFP in responders compared to controls were observed but were not clinically significant, supporting recovery of hepatic function post-treatment^31,32^. Non-responders, however, exhibited persistently elevated ALT and AST^33^, as well as AFP^34–36^, indicating ongoing hepatocellular injury and risk of progression to fibrosis or malignancy.

The distribution of LDL-R (exon 8 C.1171 G/A) gene polymorphisms (AA, GA, and GG genotypes) significantly differed between the responder and non-responder groups following antiviral therapy. Among the responders group, the AA genotype was predominant (61.7% ) followed by GA (31.6%) and GG (6.7%). The higher prevalence of the AA genotype among responders suggests a potential favorable association with antiviral therapy response, rather than a definitive predictive or causal effect^37–39^. Among the non-responders, the GG genotype was most frequent (76.7%), while GA and AA were observed in 20% and 3.3% of patients, respectively. The GG genotype was markedly associated with non-response, indicating it may contribute to resistance or suboptimal viral clearance^40,41^. The association between LDL-R (exon 8 C.1171 G/A) genotypes and treatment response was statistically highly significant. These results are consistent with emerging evidence that LDL-R (exon 8 C.1171 G/A) genetic variants influence HCV pathogenesis, as LDL-R plays a role in viral entry and hepatic lipid homeostasis. Patients carrying the GG genotype may exhibit altered LDL-R expression or function, which could be associated with reduced antiviral effectiveness or persistent viral infection. Further investigation into the mechanistic implications of LDL-R (exon 8 C.1171 G/A) polymorphisms is warranted. Importantly, these results should be interpreted as associations rather than evidence of predictive or causal relationships and the potential clinical utility of LDL-R genotyping requires validation in larger studies.

A notable variation was identified in the distribution of LDL-R (exon 8 C.1171 G/A) gene polymorphisms (AA, GA, and GG genotypes) between the responder group and the control group (*n* = 50). The AA genotype was predominant (61.7%), followed by GA (31.6%) and GG (6.7%). The control group indicated a more balanced distribution: 32% AA, 42% GA, and 26% GG. The notably higher prevalence of the AA genotype among responders suggests it may confer a beneficial effect in achieving an SVR to sofosbuvir-based therapy^42^. This advantage is attributed to genotype-related differences in LDL-R expression or function, potentially facilitating enhanced viral clearance and improved immune modulation. The GG genotype, more frequent in healthy controls than in responders, may be associated with a less favorable therapeutic response or may exert a neutral effect on treatment efficacy^43^. The study compared the distribution of LDL-R (exon 8 C.1171 G/A) gene polymorphisms (AA, GA, and GG genotypes) in patients unresponsive to sofosbuvir-based antiviral therapy versus a healthy control group. Among non-responders, the GG genotype was predominant, found in 76.7% of patients, followed by GA (20%) and AA (3.3%). The control group indicated a more balanced distribution: 32% AA, 42% GA, and 26% GG. The overrepresentation of the GG genotype in non-responders suggests a strong association with poor treatment response due to the change in LDL-R expression, a function linked to this variant, or a decrease in the effectiveness of antiviral drugs. The markedly elevated frequency of the AA genotype among responders suggests it may confer a genetic advantage in achieving a favorable treatment response^44^. This may be due to enhanced LDL-R expression or function, which could improve viral entry inhibition, drug efficacy, or immune modulation. The GG genotype, more common in controls than in responders, may be associated with decreased therapeutic effectiveness^45^. These results are consistent with emerging evidence that LDL‑R (exon 8 C.1171 G/A) genetic variants influence HCV pathogenesis, as LDL‑R has been implicated in aspects of the viral life cycle including entry and replication in host cells. Previous studies showed that modulation of LDL‑R expression alters HCV infectivity and replication, suggesting its functional relevance to viral attachment and propagation in hepatocytes^52^.

This variant might result in altered receptor activity, potentially leading to persistent HCV infection or diminished antiviral response. These results support growing evidence that genetic variation in LDL-R influences individual responses to antiviral therapy. Given LDL-R’s crucial role in mediating HCV entry into hepatocytes, such polymorphisms could directly impact infection dynamics and treatment success^46^. The low frequency of the AA genotype among non-responders, compared to its higher prevalence in controls, supports its potential protective role in enhancing treatment outcomes^47^. Nevertheless, these findings should be interpreted with caution and further well-designed studies involving larger cohorts are required to comprehensively evaluate the influence of additional host genetic, viral, and clinical factors on antiviral treatment response, as well as to validate the clinical applicability of LDL-R (exon 8 C.1171 G/A) polymorphism as a predictive biomarker.

Our analysis showed that among the responders, 20 samples were identified as HCV genotype 4. In contrast, among the non-responders, 10 out of 20 samples were genotype 4, while 3 were genotype 1, 4 were genotype 2, and 3 were genotype 6. The emergence of the unique HCV genotype 4 epidemic in Egypt is linked to the parenteral anti-schistosomal treatment campaigns that began in the 1940s and continued until oral therapy was introduced in the 1980s^48^. Previous studies have indicated a low degree of genetic heterogeneity among HCV genotypes obtained from Egypt, particularly in the Nile Delta and Nile Valley regions, where parenteral programs were implemented most extensively. Ray et al.^49^ examined 190 blood samples from patients across 15 geographically diverse governorates (excluding Sharkia) and found that the Egyptian HCV epidemic comprises multiple lineages of genotypes 1 and 4, including subgenotypes 4a, 4o, and 1 g^50^.

Our findings regarding LDL‑R (exon 8 C.1171 G/A) polymorphisms and antiviral therapy response are consistent with prior evidence in Egyptian patients with chronic HCV genotype 4. For instance, a study of 657 Egyptian patients with genotype 4 demonstrated significant differences in LDL‑R exon 8 C.1171 G > A genotype distributions between responders and non-responders, showing that carriers of the A allele had a higher likelihood of treatment success, emphasizing the role of this polymorphism in therapy outcomes^53,54^. Although earlier studies mainly investigated interferon-based regimens, they similarly highlight the influence of host genetic variation on treatment response, supporting the relevance of our results in the context of sofosbuvir-based therapy. Furthermore, studies of other genetic polymorphisms in Egyptian HCV genotype 4 cohorts, including IL‑28B variants, have confirmed that host genetics are key determinants of sustained virological response^55^. Together, these findings indicate that LDL‑R exon 8 C.1171 G/A, along with other host genetic factors, contributes significantly to inter-individual differences in antiviral therapy outcomes in Egyptian HCV genotype 4 patients. Our study extends these observations to modern direct-acting antiviral regimens, providing further evidence for the potential utility of genetic profiling in optimizing treatment strategies for this population.

### Study limitations

This study has several limitations. The relatively limited sample size used for genetic analysis was partly related to the technical complexity and financial considerations associated with molecular genotyping. Additionally, the study population was restricted to Egyptian patients, which may limit the generalizability of the findings to other populations. As an observational study, causal relationships between LDL-R polymorphisms and antiviral treatment response cannot be established. Future studies with larger, multicenter cohorts and additional host and viral variables are warranted to further elucidate the determinants of treatment response in chronic HCV infection.

## Conclusion and future perspectives

In conclusion, our study highlights the significant association between LDL-R (exon 8 C.1171 G/A) genetic polymorphisms and the response to antiviral therapy in Egyptian patients with HCV infection. The results indicated that the AA genotype of LDL-R (exon 8 C.1171 G/A) was predominant among responders to sofosbuvir-based antiviral therapy (61.7%), while the GG genotype was more prevalent in non-responders (76.7%). These findings suggest that LDL-R (exon 8 C.1171 G/A) genetic variants may be associated with treatment, potentially through mechanisms potentially affecting viral entry, lipid metabolism, or immune modulation. The high response rate (91.8%) observed in this cohort underscores the effectiveness of sofosbuvir-based regimens in HCV treatment. However, the identification of LDL-R (exon 8 C.1171 G/A) polymorphisms as a potential predictive biomarker could enhance personalized therapeutic strategies, particularly for patients at risk of suboptimal treatment response. The study reinforces the clinical relevance of monitoring liver function biomarkers such as AFP and AST, which were significantly elevated in non-responders, indicating unresolved liver injury. Overall, LDL-R (exon 8 C.1171 G/A) genotyping may serve as a valuable tool for optimizing HCV treatment protocols, particularly in resource-limited settings.

Future studies should involve larger, multicenter cohorts with diverse ethnic backgrounds to validate the observed associations and improve generalizability. Integrating LDL-R (exon 8 C.1171 G/A) genotyping with other host genetic, viral, and clinical factors may provide a more comprehensive understanding of determinants of antiviral treatment response. In addition, functional studies are warranted to elucidate the biological mechanisms underlying the influence of LDL-R polymorphisms on HCV entry and antiviral efficacy, thereby supporting their potential role in personalized treatment strategies.

## Data Availability

The sequence data generated and/or analysed during the current study are available in the DDBJ repository (INSDC member) under accession number LC909039.
